# Mild Encephalopathy With Reversible Splenial Lesion (MERS) in a Child With Influenza

**DOI:** 10.7759/cureus.50838

**Published:** 2023-12-20

**Authors:** Yeka W Nmadu, Mindy M Le, Oluwafemi G Aremu, Kuo Y Chen, Adam S Rappoport

**Affiliations:** 1 Pediatrics, University of Florida College of Medicine, Jacksonville, USA; 2 Pediatrics, University of Florida College of Medicine, Gainesville, USA; 3 Cardiovascular Center, University of Florida Health, Jacksonville, USA; 4 Pediatric Neurology, Nemours Children's Health, Jacksonville, USA

**Keywords:** mers, central nervous system, splenium of the corpus callosum, influenza b, reversible encephalopathy

## Abstract

Mild encephalopathy with reversible splenial lesion (MERS) is a rare clinical-radiological syndrome with a favorable prognosis that typically presents with central nervous system symptoms such as altered mental status, delirious behavior, seizures, muscle weakness, ophthalmoplegia, and headache. The diagnosis of MERS is based on a constellation of central nervous system symptoms within one week of fever, a lesion in the splenium of the corpus callosum, and complete resolution without sequelae. Both clinical and imaging findings generally resolve within a few months. Treatment is largely supportive care and/or treatment of the primary cause.

## Introduction

Mild encephalopathy with reversible splenial lesion (MERS) is a reversible clinical-radiological syndrome that is observed in patients with encephalitis or encephalopathy due to infection (most commonly viral infections), metabolic disorders, abrupt cessation of steroid therapy, and/or use/discontinuation of antiepileptic drugs [1-3]. It was first described by Tada et al. [4] and noted to have a favorable prognosis. MRI is required for diagnosis [4,5]. Here, we report the case of an eight-year-old with MERS following an influenza B infection.

## Case presentation

**Figure 1:**
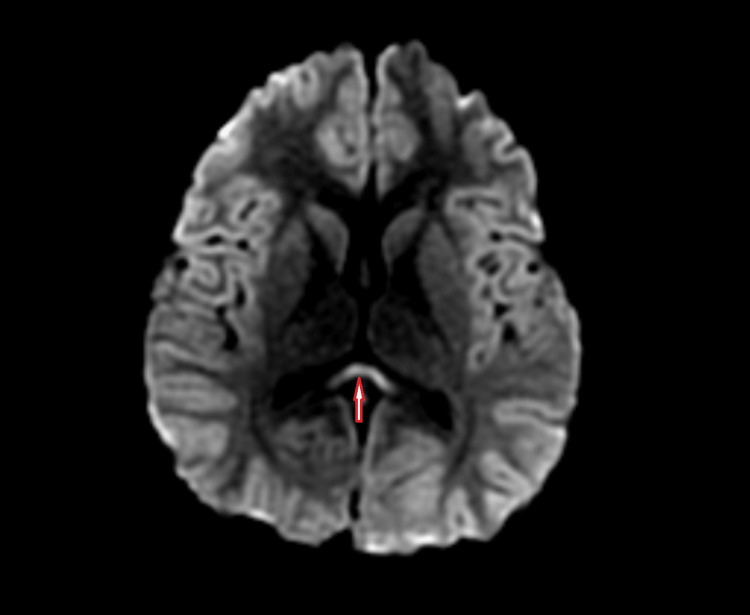
Neuroimaging showing hyperintensity involving the splenium of the corpus callosum (red arrow), aka the “boomerang sign”

A previously healthy, eight-year-old female presented to the ER with a headache and fever for four days and visual hallucinations for three days. Symptoms started with malaise and subsequently headache, fatigue, and fever up to 103 F. By the second day, she had developed visual hallucinations, crying spells, and episodes of decreased responsiveness. Upon her presentation to the ER, her vital signs were normal except for tachycardia. Her Glasgow Coma Scale score was 15. There were no meningeal signs and the rest of the exam was unremarkable including a normal, non-focal neurological exam. Labs showed leucopenia with neutrophilia, hyponatremia, and a slight elevation in partial thromboplastin time. Urinalysis showed ketonuria, nine white blood cells, and small leukocyte esterase. Cerebrospinal fluid cell count, protein, and glucose were within the normal range. The urine drug screen and rapid strep were negative. A non-contrast head CT was normal. She was diagnosed with a urinary tract infection and confusion in the setting of a fever and discharged home on antibiotics. She appeared stable on the third day of illness except for a few episodes of erratic behavior. By day four, her parents noticed conjunctival erythema, she was lethargic and yelled out in her sleep, had incoherent speech, and had episodes of interchanging crying and laughing. She was brought back to the ER. Again, vitals were stable except for tachycardia. Physical examinations, including neurologic exams, were within normal limits. She had worsening leucopenia with neutropenia. The complete metabolic profile was normal and the initial urine culture only isolated urogenital flora. All cultures obtained at the first ER visit yielded no growth. MRI showed a signal abnormality in the splenium of the corpus callosum with restricted diffusion, findings concerning acute infarct (Figure 1).

Magnetic resonance angiography (MRA) and magnetic resonance venography (MRV) were ordered as part of the institutional stroke protocol. MRA of the head and neck showed a slightly decreased branching pattern within the distal branches of the right posterior cerebral artery. The MRV was normal. An electroencephalogram (EEG) was ordered to rule out the epileptic etiology of her symptoms and showed bilateral posterior quadrant epileptiform discharges over the parietal-temporal area, which were bisynchronous with the midline parietal electrode. The abnormal EEG was indicative of focal cerebral dysfunction with concern for structural abnormality over the parietal-temporal head area, suggesting localization-related epilepsy. No seizures were captured. Continuous EEG monitoring showed improvement in her EEG tracing and resolution of epileptiform discharges. Ultimately, she tested positive for influenza B and was diagnosed with MERS. She remained stable and was discharged home to follow up with her primary care provider.

## Discussion

MERS is the most reported reversible splenial lesion syndrome [6]. Although MERS has been reported in different regions of the world, most are reported in Eastern Asian countries [7]. Like our patient, a few cases have been reported in North America [1,4,8].

In children, the presentation of MERS is non-specific but can present with central nervous system symptoms such as altered mental status, delirious behavior, seizures, muscle weakness, ophthalmoplegia, and headache [1,3]. A multicenter study from Japan identified that the most common neurological symptom (identified in 54% of patients) is delirious behavior, which was also seen in our patient [9]. Subsequent studies have identified seizures as the most common presenting symptom [10]. Different viruses have been associated with MERS including influenza virus, human herpesvirus-6, rotavirus, and mumps; our patient tested positive for influenza B [11]. The reported median age of onset of MERS is five years of age with a standard deviation of 5.6 ± 3.7 years; our patient fits into this age bracket [11].

The diagnosis of MERS is based on a constellation of five clinical criteria [11]: (1) the onset of central nervous system symptoms within one week of fever; (2) complete resolution without sequelae, mostly within 10 days after onset of symptoms; (3) a high-signal intensity lesion in the splenium of the corpus callosum; (4) the lesion may involve the entire corpus callosum and the cerebral white matter in a symmetrical lesion; and (5) the lesion disappears within a week, with neither residual changes nor atrophy. The characteristic splenial lesion is slightly hyperintense on diffusion-weighted sequences, with decreased signal intensity on apparent diffusion coefficient maps and diffusion restriction without contrast enhancement at post-contrast images during the acute period of the disease [3,10]. This lesion has been described as the “boomerang sign,” as seen in our patient’s MRI shown in Figure 1 [12]. Two forms of MERS, namely type 1 and type 2, have been described. MERS type 1 has an isolated lesion in the splenium of the corpus callosum, while MERS type 2 presents with extensive white matter and/or entire callosal lesions [9]. EEG findings in MERS include diffuse slow waves, occipital slow waves, and diffuse high-voltage slow waves [3]. There have also been reports that MERS may be related to significantly higher theta band power in the frontal and center of the parietal lobes on the EEG [13]. The index case had low-amplitude spikes in the bilateral posterior quadrants.

The exact pathophysiology of MERS is unknown but various mechanisms have been proposed. Possible pathophysiology includes intra-myelinic edema, a transient inflammatory infiltrate, vascular redistribution, arginine vasopressin suppression, and interstitial edema in tightly packed fibers [4,9,14]. The lack of a causative agent in the cerebrospinal fluid cultures of most patients suggests an indirect mechanism, such as immune-mediated, and the release of myelin-specific neurotoxins could cause inflammatory infiltrates resulting in cerebral edema [10,15]. It has also been thought that hypotonic hyponatremia could lead to the entry of water into the brain and, hence, intra-myelinic edema, causing the transient reduced diffusion seen on MRI [9]. As seen in our patient, hyponatremia is common in patients with MERS [9,15].

Treatment is largely supportive care and/or treatment of the primary cause. Our patient's clinical condition improved with supportive care. There are reports of the use of methylprednisolone pulse therapy and intravenous immunoglobulin in some patients [16]. Both clinical and imaging findings generally resolve within a few months. Most commonly, patients with MERS type 1 were generally reported to recover completely both clinically and on imaging, while type 2 lesions might persist for months and some patients have neurological sequelae [5,10,17].

## Conclusions

Although it presents with concerning symptoms, MERS is most often associated with a favorable clinical course. It is commonly associated with viral infections. Early diagnosis is essential to avoid unnecessary investigations and interventions. A high index of suspicion is required to ensure optimal care and provide reassurance to the caregivers.
